# Pembrolizumab-Induced Ocular Myasthenic Crisis

**DOI:** 10.7759/cureus.9192

**Published:** 2020-07-14

**Authors:** Christian J Lorenzo, Haley Fitzpatrick, Victoria Campdesuner, Justin George, Natalia Lattanzio

**Affiliations:** 1 Internal Medicine, Sarasota Memorial Hospital, Florida State University College of Medicine, Sarasota, USA; 2 Pharmacy, Sarasota Memorial Hospital, Sarasota, USA

**Keywords:** pembrolizumab, keytruda, myasthenia gravis, ocular, myasthenic crisis, anti-pd-1, diplopia, ptosis, immune checkpoint inhibitor

## Abstract

Pembrolizumab, one of many novel immune checkpoint inhibitors (ICPi), is a monoclonal antibody that enhances immunity against cancer cells. Extensive escalation in immune activity predisposes to unsought immune-related adverse events. Due to progressive mesothelioma, a 67-year-old man was referred to the research unit and enrolled in a clinical trial with a cluster of differentiation (CD) 27 chemotherapeutic agent. He began crossover treatment and received just two doses of pembrolizumab, 33 and 16 days prior to admission. He subsequently presented to the emergency department with three days of acute onset severe diplopia and a drooping left eye. Acetylcholine receptor (AChR) antibodies returned positive at 13.9 nmol/L, and a diagnosis of ocular myasthenia gravis (OMG) was made. During his hospitalization, the patient was managed with methylprednisolone 80 mg intravenously daily, with conversion to prednisone 60 mg by mouth daily at time of discharge. Neuro-ophthalmology consultation was sought in the outpatient setting, and the patient was started on pyridostigmine. He was readmitted two weeks later with symptoms of progressive diffuse weakness, unsteady gait, and dysphagia, all in the setting of persistent diplopia. Intravenous immunoglobulin (IVIG) was promptly initiated, in addition to the pyridostigmine previously initiated in the outpatient setting. Unfortunately, after three IVIG treatments, the patient had experienced little improvement in his symptoms, and therefore elected hospice care. Although ICPis have revolutionized the management of a multitude of malignancies, recognition of immune-related adverse events is of critical importance.

## Introduction

Pembrolizumab, one of many novel immune checkpoint inhibitors (ICPi), is a highly selective humanized immunoglobulin 4 (IgG4) monoclonal antibody that enhances immunity against cancer cells. This is achieved by inhibition of the programmed death (PD) pathway. During cell-mediated immune encounters, tumor cell PD-1 ligands (PDL) are appreciated by PD receptors on the surface of T-cells. This interaction will halt T-cell defenses, allowing the cancer cells to continue to proliferate. Pembrolizumab and nivolumab (a similar monoclonal antibody) act by inhibiting PD-1 ligation, permitting restoration in T-cell-driven anti-tumor response [1]. Unfortunately, extensive escalation in immune activity predisposes to unsought immune-related adverse events. Myasthenia gravis (MG) is a neurological disorder that affects the neuromuscular junction causing muscle weakness and fatigability in a plethora of clinical presentations [2]. MG is a relatively rare adverse event associated with both pembrolizumab and nivolumab. Among 23 reported cases of ICPi-associated MG summarized in 2017, 72.7% were de novo presentations, 18.2% were exacerbations of pre-existing MG, and 9.1% were considered to be exacerbations of subclinical MG [3]. Additionally, MG very rarely may manifest with symptomatology exclusive to the ocular muscles, known as ocular myasthenia gravis (OMG). Although there have been multiple post-surveillance case reports of patients receiving anti-PD-1 therapy presenting with new or exacerbated MG, to date, there have only been eight reported cases of anti-PD-1 associated OMG; five of which were a direct result of pembrolizumab [4-9]. A case of pembrolizumab-induced OMG is described.

## Case presentation

A 67-year-old male with a medical history of malignant mesothelioma presented to the emergency department with three days of acute onset severe diplopia. He had associated frontal headache, blurred vision, and horizontal binocular diplopia. Symptoms were alleviated when he closed either eye. He also noticed drooping of the left eye. He notably denied any focal deficits or dysarthria. The patient previously had been receiving chemotherapy for the past 10 years including cisplatin, pemetrexed, and gemcitabine. Prior to admission, due to progression of his mesothelioma, he was referred to the research unit and was enrolled in a clinical trial with a novel cluster of differentiation (CD) 27 chemotherapeutic agent. He began crossover treatment and received a total of just two pembrolizumab doses, 33 and 16 days prior to admission. Physical examination was pertinent for a visual acuity of 20/20 in the right eye and 20/25 in the left eye. Pupils were equal, round, and reactive to light. Examination of extraocular movements revealed a right eye abduction deficit of approximately 25% and a 100% abduction deficit in the left eye. He denied diplopia on primary gaze. On rightward gaze, there was a horizontal diplopia with an oblique component appreciated. There was ptosis of the left eye present. Fundoscopic examination revealed no papilledema. The patient had normal muscle bulk and tone with 5/5 strength in all four extremities. 

Sedimentation rate was found elevated at 37 mm/hr. Lumbar puncture was performed, which yielded 15 cc of cerebral spinal fluid (CSF) with an opening pressure measured at 17 cmH_2_O. Cytology of the fluid was grossly unremarkable, as were cultures. Of the CSF studies, glucose was 64 mg/dL, protein was 41 mg/dL, and Venereal Disease Research Laboratory test (VDRL) was negative. CT angiogram of the head and neck revealed no occlusion or flow-limiting stenosis. Acetylcholine receptor (AChR) antibodies returned positive at 13.9 nmol/L. While hospitalized, the patient, with an admission weight of 86.1 kg, was treated with methylprednisolone 80 mg intravenously daily, with conversion to prednisone 60 mg by mouth daily at the time of discharge. However, several days later, the patient experienced progressive diffuse weakness and unsteady gait, in addition to persistent diplopia. Neuro-ophthalmologic consultation was sought in the outpatient setting, and he was started on pyridostigmine. He was readmitted two weeks later with worsening neurological symptoms of paresis and dysphagia. Intravenous immunoglobulin (IVIG) was initiated for a planned five days of treatment, in addition to his pyridostigmine. After three days of therapy with little improvement in his symptoms, the patient elected hospice care, which was coordinated.

## Discussion

The neuromuscular junction is a synaptic communication between nerve endings and a muscle. When an action potential propagates down a motor nerve to the nerve terminal, acetylcholine (ACh) is released from 150-200 vesicles, also known as quama, from the presynaptic nerve fiber. The subsequent ligation of ACh to AChR can depolarize the postsynaptic membrane, allowing for the muscle to contract. The pathophysiology of MG most commonly includes the creation of antibodies against the AChR, which decreases the number of available AChRs at the postsynaptic muscle membrane. Therefore, despite adequate ACh release, end-plate action potentials fail to trigger muscle action potentials resulting in muscular paresis [2]. A large population of patients with systemic MG also have ocular involvement that can occur either initially or later during the clinical course of the disease [10].

OMG is a subtype of MG where muscular fatigue is clinically isolated to the extraocular muscles (EOMs), levator, and orbicularis oculi. Signs and symptoms of OMG include ptosis and diplopia, which are present in over 50% of systemic MG. The majority of OMG patients (50%-80%) go on to develop generalized MG. OMG can mimic cranial nerve pathology, gaze palsies, internuclear ophthalmoplegia, blepharospasm, and stroke. All of these etiologies were in the differential diagnosis of this patient. Serum antibodies are present in 99% of patients with generalized MG but only 40%-70% of patients with OMG. EOMs develop tension quicker and have a higher frequency of synaptic firing than muscle groups of the upper and lower extremities. As a result, they are more vulnerable to fatigue and exhaustion. Additionally, tonic muscle fibers are critical to sustainment of ocular gaze [10].

Lower temperatures have been hypothesized to decrease the activity of acetylcholinesterase (AChE), which is an enzyme present within the synaptic folds responsible for hydrolyzing ACh. If a patient has ptosis, application of an ice pack over the ptotic eye often will result in clinical improvement of ptosis. This is hypothesized to be secondary to decreased AChE activity, in addition to less depletion of quanta occurring secondary to decreased temperature. The ice test is not necessarily diagnostic but can raise the suspicion of MG [1,2]. The sleep test evaluates for resolution of ptosis or ophthalmoparesis immediately after a 30-minute period of sleep [10].

In a patient with typical symptomatology and physical exam findings of OMG, the diagnosis can be solidified with a positive AChR antibody titer. If titers are negative, as they are in about 30%-77% of cases, electromyography with repetitive nerve stimulation or single-fiber electromyography is necessary in order to establish a diagnosis. If the above studies are negative, MRI of the brain and lumbar puncture with CSF analysis may be warranted to exclude further inflammatory or structural pathology of the central nervous system [10]. 

Adverse events are unfavorable signs, symptoms, or diseases which are temporally associated with the use of a medical treatment or procedure that may or may not be considered related to said treatment or procedure. A grading system, developed by the Cancer Therapy Evaluation Program, spanning from grade 1-5 evaluates the severity of these adverse events. Grade 1 indicates a mild event in which intervention is not indicated. Grade 2 is deemed as moderate, grade 3 as severe, grade 4 as life-threatening, and grade 5 as resulting in death; all warranting implementation of intervention.

In regard to the management of anti-PD-1 associated MG, there is no grade 1 toxicity. All grades warrant a thorough diagnostic workup and intervention given the potential risk of progression of MG-induced respiratory insufficiency. In grade 2 MG (adverse event), it is recommended to hold the anti-PD1 ICPi which may be resumed if symptoms resolve. Neurology consultation should be sought, and the patient should be offered pyridostigmine with an initial dose of 30 mg by mouth three times daily. The dose can gradually be increased to a maximum daily dose of 480 mg divided in four doses. In all grades of MG, systemic steroids remain the mainstay treatment (e.g., prednisone 1-1.5 mg/kg by mouth daily). Notably, if the patient presents with grade 3 or 4 MG, the anti-PD1 ICPi must be permanently discontinued. The patient should be admitted potentially to the intensive care unit and again, neurology consultation is warranted. IVIG 2 g/kg administered over five days versus plasmapheresis for five days is currently recommended in addition to corticosteroid therapy. Frequent pulmonary function assessment should be offered due to the risk of respiratory compromise seen in systemic MG [11].

## Conclusions

This case demonstrates an infrequent and uniquely characteristic complication of pembrolizumab presenting in the form of ptosis, oculomotor paresis, and diplopia. A diagnosis of OMG was confirmed with AChR antibody positivity. A comprehensive diagnostic workup for all grades of ICPi-induced MG is warranted, including laboratory, imaging, and potential pulmonary function testing. As the majority of OMG patients do, this patient eventually progressed to systemic MG requiring not only corticosteroid therapy but also IVIG. Although ICPis have revolutionized the management of a multitude of oncologic malignancies, recognition of immune-related adverse events is of critical importance.
